# The Role of Hyaluronic Acid in Alveolar Ridge Preservation: A Systematic Review of Its Biological and Regenerative Potential According to PRISMA Guidelines and the Cochrane Handbook

**DOI:** 10.3390/biomedicines13020451

**Published:** 2025-02-12

**Authors:** Vincenzo Ronsivalle, Simona Santonocito, Roberta Giudice, Salvatore Bocchieri, Simone Didomenico, Marco Cicciù

**Affiliations:** 1 Department of Biomedical and Dental Sciences, Morphological and Functional Images, University of Messina, G. Martino Polyclinic, 98124 Messina, Italy; vincenzo.ronsivalle@hotmail.it (V.R.); salvo.bocchieri@gmail.com (S.B.); 2 Department of General Surgery and Surgical-Medical Specialties, School of Dentistry, University of Catania, 95124 Catania, Italy; robertagiudice@virgilio.it (R.G.); didomenicosi@gmail.com (S.D.); marco.cicciu@unict.it (M.C.)

**Keywords:** hyaluronic acid, alveolar ridge preservation, bone regeneration

## Abstract

**Objectives**: This systematic review evaluates the biological activity and regenerative potential of hyaluronic acid (HA) in alveolar ridge preservation (ARP) following tooth extraction, assessing its efficacy in reducing bone resorption and promoting bone regeneration when combined with xenografts. **Methods**: A comprehensive search was conducted across PubMed, Scopus, Web of Science, and Lilacs databases, adhering to PRISMA guidelines. Studies from 2012 to December 2024 involving human participants were included based on a PECO framework. Four studies met the inclusion criteria, with data extracted and analyzed for clinical and histological outcomes. The risk of bias was assessed using the ROBINS-E tool. **Results**: The included studies demonstrated that HA combined with xenografts significantly reduced post-extraction bone resorption compared to controls. HA-enhanced grafts showed superior radiographic and histological outcomes, including increased bone density and reduced graft shrinkage. While one randomized controlled trial found no significant differences in wound healing or patient-reported outcomes between HA and control groups, other studies reported improved bone formation and graft stability with HA. Variability in study design and sample size was noted, with a generally moderate to high risk of bias in some studies. **Conclusions**: The evidence supports the beneficial role of HA as an adjuvant in ARP procedures, enhancing bone regeneration and limiting resorption. However, further research with larger samples and standardized methodologies is required to confirm these findings and optimize clinical protocols.

## 1. Introduction

Following the tooth extraction, the alveolar socket undergoes resorption due to the healing process [1]. The healing of the extraction socket occurs through a sequence of events, starting with the formation of a blood clot, followed by the creation of a highly vascularized provisional matrix, and culminating in osteogenesis [2]. The resorption occurs mainly in the first 8 weeks after the tooth is extracted, and the original ridge width can undergo a reduction of up to 50% of its first size [3]. The resorption occurs, above all, on the buccal part of the ridge, and this conditions the prognosis when the treatment plan is based on a prosthetically driven implant [4]. For this reason, the management of the ridge volume is obligatory, especially in the anterior sector [5]. In non-molar regions, this results in reductions in the ridge measuring 2.73 mm horizontally, 1.71 mm vertically at the midfacial aspect, and 1.44 mm at the mid-lingual area [6]. Some techniques have been proposed to reduce this loss. These techniques are ARP, guided bone regeneration, and onlay bone block [7,8]. ARP is a procedure that involves the use of biomaterials placed inside the socket after the tooth extraction to limit post-extraction remodeling [9]. A crucial element in ARP is the soft tissue barrier that safeguards the underlying bone replacement graft [10]. In these years, several growth factors acting as biological adjuvants have been tested to find out which one promotes the healing process and decreases the physiological post-extractive bone resorption [11]. There are several types of bone grafting materials on the market, but the most used are allografts and xenografts [7]. Demineralized bovine bone material has been proven to be biologically inert and osteoconductive [7]. Xenografts offer adequate bone volume maintenance, but their biggest limits are the healing period and the amount of new bone regeneration [12]. ARP aims to promote new bone formation within the socket of the extracted tooth [9], although there is no agreement on the best approach to managing soft tissues in the context of ARP [13]. ARP is often achieved using a collagen matrix to close the socket. A recent clinical investigation comparing ARP with a free gingival punch graft harvested from the palate to ARP with a collagen matrix revealed that the latter resulted in significantly less scarring [14]. An advantage of a collagen matrix is that it eliminates the need for a second surgical site for graft harvesting [15]. Vascularization and epithelialization of the socket seal grafting material are essential for achieving complete wound healing quickly, which helps reduce bacterial contamination of the underlying bone replacement graft [7]. In this context, wound-healing agents can significantly accelerate the healing process, ultimately aiming to improve ARP [9]. High-molecular-weight hyaluronic acid (HA) shows promise as a wound-healing agent for this purpose due to its bacteriostatic, anti-inflammatory, and immunosuppressive properties [16]. The application of HA in the oral cavity has been widely researched. In vitro studies show that HA promotes the proliferation and migration of human oral fibroblasts and periodontal ligament cells [17,18,19]. More specifically, HA also stimulates mesenchymal cell proliferation, angiogenesis in situ, and growth factor attraction [20,21]. The clinicians aim to place dental implants as early as possible following the tooth extraction [11].

Therefore, the aim of this review is to evaluate the biological activity of HA gel as an adjuvant in the xenograft to improve bone regeneration properties and limit bone post-extraction resorption.

## 2. Materials and Methods

### 2.1. Eligibility Criteria

All documents were selected based on the following Population, Exposure, Comparator, and Outcomes (PECO) model:

- (P) Participants consist of human subjects;
- (E) The exposure consisted of tooth extraction;
- (C) The comparison consisted of subjects treated with and without HA;
- (O) Outcome: The usefulness of hyaluronic acid in reducing the resorption after tooth extraction.

Research papers were only taken into consideration for inclusion if they provided conclusive data once the intervention was finished. The exclusion criteria were as follows:

(1) Articles that did not comply with PECO, (2) duplicate articles, (3) studies involving animals, (4) review articles, (5) studies written in a language different from English, (6) full-text unavailability, and (7) systemic diseases.

### 2.2. Search Strategy

We used a systematic search strategy to search the PubMed, Scopus, Web of Science, and Lilacs databases for publications published from 2012 to the 2nd of December 2024. The search strategy is indicated in Table 1.

Additionally, a manual search for previous systematic reviews on the same topic was conducted. This systematic review followed the Preferred Reporting Items for Systematic Reviews (PRISMA) guidelines 2020 and the Cochrane Handbook for Systematic Reviews of Interventions. PROSPERO’s (International Prospective Register of Systematic Reviews) lists the systematic review protocol under the provisional accession number CRD635808.

### 2.3. Data Extraction

Two reviewers (S.D. and V.R.) independently extracted the data from the studies using tailored data extraction on an Excel sheet. A third reviewer reached a consensus on the case of disagreement (M.C.)

The following data were extracted (Table 2): (1) first author, (2) year of publication, (3) patients’ age, (4) evaluation of hyaluronic acid and alveolar ridge preservation and results, (5) clinical relevance.

### 2.4. Quality Assessment

Two reviewers (S.S. and V.R.) used the Robins-E technique to evaluate the risk of bias. Any disagreement was handled with a third reviewer (M.C.) until a consensus was obtained.

In this review, the Risk of Bias in Non-randomized Studies of Interventions (ROBINS-E) tool was employed to assess potential biases in the included studies. This tool offers a structured framework to evaluate bias across seven domains: confounding, participant selection, intervention classification, deviations from intended interventions, missing data, outcome measurement, and selection of reported results.

Two or more trained reviewers independently evaluated each study, following ROBINS-E guidelines. This process ensured objectivity and consistency in the assessments. The ROBINS-E evaluation provided a comprehensive analysis of potential biases, highlighting both the strengths and limitations of the evidence base. This, in turn, informed the interpretation of results and supported more reliable conclusions based on the available data.

## 3. Results

### 3.1. Study Characteristics

**Figure 1:**
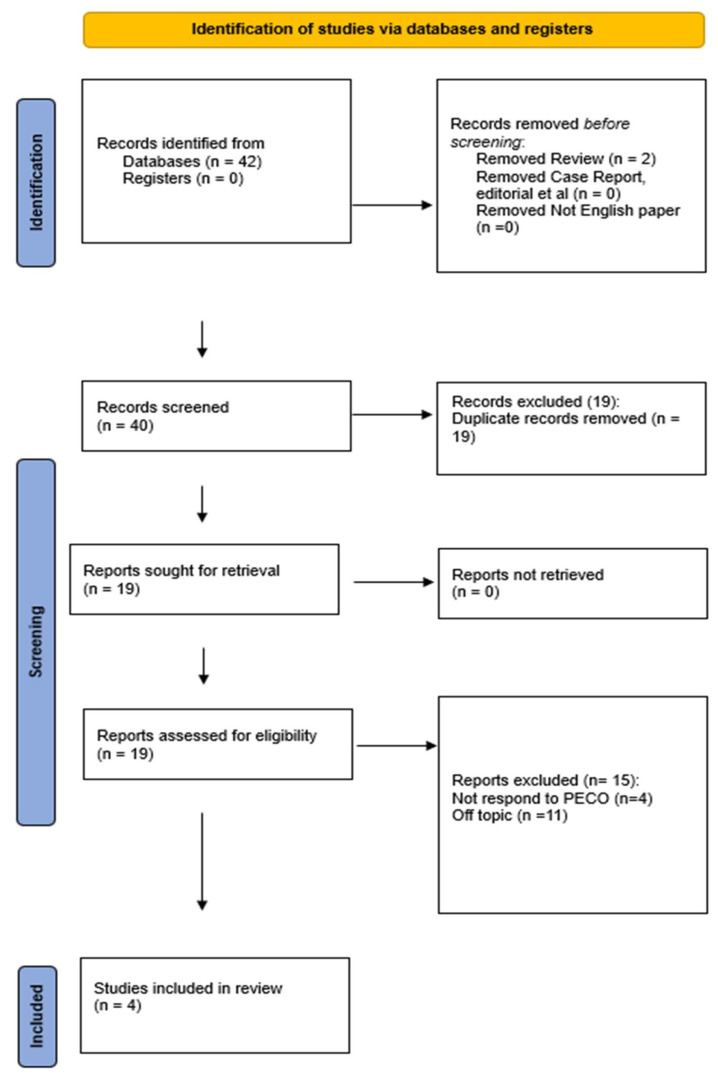
PRISMA flowchart of the included study.

A total of 42 articles were selected after using four search engines. The Boolean operator “NOT” was used to exclude articles written in a different language from English based on exclusion criteria. Two articles were excluded by using the exclusion criteria “review articles”. During the initial screening step, 40 articles were considered. Due to their duplication, 19 articles were removed. Then, 19 articles were assessed for eligibility. A total of 11 of these were excluded because they were off-topic, and 4 did not respond to PECO. Hence, four articles were included in this review, as illustrated by the PRISMA 2020 flowchart (Figure 1).

### 3.2. Main Findings

The retrospective study by Kloss et al. [23] evaluated the effectiveness of ridge preservation using allogeneic bone substitutes with and without HA in 40 patients. The addition of HA significantly improved outcomes, reducing vertical bone loss (−0.19 ± 0.51 mm in the HA group vs. −0.82 ± 0.95 mm, *p* = 0.011) and graft shrinkage (10.3% vs. 16.9%, *p* = 0.038). Furthermore, bone density was higher in the HA group after 4 months (211.03 ± 67.35 HU vs. 132.66 ± 48.85 HU, *p* = 0.004). Both groups demonstrated a 97.5% implant success rate. The incorporation of HA enhanced graft stability, reduced resorption, and supported better bone regeneration through its viscoelastic properties and effects on osteogenesis and wound healing. This highlights its potential to optimize ARP procedures.

Husseini et al. [22] retrospective study compared ridge preservation with allogeneic bone grafts enriched with hyaluronic acid (AlloHya) and without it (Allo) in 40 patients. Results revealed significant benefits of AlloHya, including reduced vertical bone loss (−0.19 mm vs. −0.82 mm, *p* = 0.011), lower graft shrinkage (10.3% vs. 16.9%, *p* = 0.038), and higher bone density after four months (211.03 vs. 132.66 Hounsfield units, *p* < 0.01). Both groups showed no differences in horizontal bone stability or implant success rates (39/40 implants successful). Hyaluronic acid likely enhanced osteogenesis and graft stability by promoting hydration, reducing resorption, and stimulating osteoblast activity. The findings highlight hyaluronic acid’s potential as an adjunct in ridge preservation to optimize clinical outcomes and improve implant integration.

The Eeckhout et al. [24] randomized controlled trial (RCT) evaluated the effects of hyaluronic acid (HA) gel as an adjunct in ARP. Forty-six extraction sites were randomly assigned to a test group (HA gel application) or a control group (no HA). Wound dimensions, soft tissue changes, bone resorption, and patient-reported outcomes were analyzed. Results indicated no significant differences between the groups in terms of wound healing, pain, alveolitis incidence, or soft tissue outcomes. Interestingly, the test group exhibited greater horizontal bone loss at coronal levels (*p* ≤ 0.025). Despite HA’s known bacteriostatic and anti-inflammatory properties, its application did not enhance wound resolution or bone preservation when used with a collagen matrix in ARP. These findings suggest that HA gel does not improve clinical outcomes in ARP and may contribute to increased bone resorption.

Abaza et al.’s [3] RCT compared injectable platelet-rich fibrin (I-PRF) and hyaluronic acid (HA) combined with bovine-derived xenografts for ARP. Thirty-six patients were assigned to three groups: I-PRF with xenografts, HA with xenografts, or xenografts alone. Outcomes were assessed radiographically, clinically, and histologically over one year. The HA group showed superior radiographic bone preservation (mean bone gain: 9.78 ± 0.87 mm) and histological bone maturity compared to the I-PRF and control groups. Histomorphometric analysis revealed that HA yielded higher newly formed bone (56.66 ± 7.35%) and lower residual graft material compared to I-PRF. I-PRF improved soft tissue thickness but underperformed in bone formation metrics. Overall, HA-xenograft combinations provided enhanced bone preservation and maturation, whereas I-PRF was more effective in soft tissue enhancement.

### 3.3. Quality Assessment and Risk of Bias

**Figure 2:**
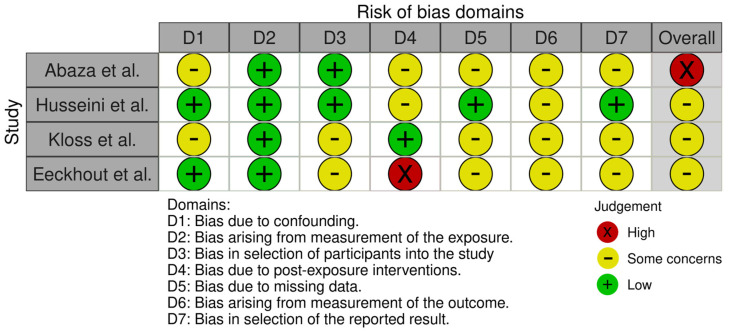
ROBINS-E tool for risk of bias [3,22,23,24].

The risk of bias was assessed using the ROBINS-E tool (Figure 2), as shown in the figure. Regarding confounding bias (D1), two studies (Husseini et al. [22] and Eeckhout et al. [24]) demonstrated a low risk of bias. Bias arising from the measurement of exposure (D2) was rated as low across all studies. Bias in the selection of participants (D3) was also consistently low in three studies, with some concerns noted by Kloss et al. [23].

For post-exposure interventions (D4), Abaza et al. [3] and Kloss et al. [23] showed some concerns, while Eeckhout et al. [24] displayed a high risk of bias. Bias due to missing data (D5) and outcome measurement (D6) was rated as having some concerns for all studies. However, bias in the selection of the reported result (D7) was low for Husseini et al. but noted as having some concerns for the remaining studies.

Overall, the studies were judged as follows: high risk of bias for Abaza et al. [3], low risk for Husseini et al. [22], and some concerns for Kloss et al. [23] and Eeckhout et al. [24]. These assessments highlight areas for improvement in study design and reporting practices.

## 4. Discussion

Some studies have shown that hyaluronic acid exhibits notable osteoinductive effects, helping to minimize bone resorption when applied in ARP procedures [25,26,27,28,29].

The findings from these studies consistently highlight the potential benefits of HA as an adjunct in ARP, particularly when combined with grafting materials. Abaza et al. [3] demonstrated that HA combined with xenografts significantly outperformed I-PRF and xenografts alone in promoting radiographic bone gain, new bone formation, and histological bone maturation. These results align with previous research by Kloss et al. [23] and Husseini et al. [22], which reported that HA improved bone density, minimized vertical bone loss, and reduced graft resorption, further emphasizing its osteoconductive and osteogenic properties.

Contrarily, Eeckhout et al. [24] reported no significant clinical advantage of HA gel application in ARP, with a surprising increase in horizontal bone loss at coronal levels. This discrepancy could stem from variations in HA formulation or application protocols, suggesting that the efficacy of HA may depend on its specific use and combination with other materials.

While I-PRF showed advantages in soft tissue enhancement, HA’s overall superior performance in bone regeneration and graft stability underscores its utility in optimizing ARP outcomes.

Several studies have demonstrated that hyaluronic acid (HA) plays a pivotal role in promoting cell adhesion and proliferation, processes that are critical for the initiation of tissue regeneration. By facilitating these cellular activities, HA contributes to the formation of a robust extracellular matrix at the graft site, which serves as a structural and biological foundation for subsequent bone growth and integration. This enhanced matrix development not only supports the stability of the graft but also optimizes the microenvironment for sustained osteogenesis and improved regenerative outcomes [30,31,32,33]. HA markedly improved the healing and regeneration of periodontal wounds in two-wall mandibular intrabony defects in an animal model [34].

The application of HA in bone regeneration highlights its versatility as both a structural and functional component in biomaterial scaffolds. HA’s inherent bioactivity is mediated through interactions with CD44 receptors, enhancing mesenchymal stem cell adhesion, proliferation, and differentiation, which are critical for osteogenesis [35,36]. Its viscoelastic properties and water retention capabilities make HA an excellent carrier for bioactive molecules, such as growth factors and drugs, optimizing bone regeneration processes [37,38]. HA-based scaffolds can be tailored as rigid or colloidal forms to suit specific clinical needs. Rigid scaffolds, often combined with calcium phosphates or bioactive ceramics, provide structural support while enhancing osteoconductivity [39,40]. On the other hand, colloidal HA-based hydrogels offer adaptability, particularly in anatomically complex regions, and enable the controlled delivery of osteoinductive factors like BMP-2, achieving sustained release and improving bone repair outcomes [41]. HA’s antimicrobial and antiadhesive properties further reduce infection risks, as shown in studies where HA coatings improved osseointegration and minimized bacterial contamination [42,43,44]. Notably, the synergistic interaction of HA with hybrid scaffolds, particularly when combined with bioactive molecules such as growth factors or other polymers with complementary properties, highlights its significant potential in driving advancements within the field of regenerative applications, enabling more efficient tissue regeneration, enhanced biological integration, and tailored solutions for complex clinical scenarios [45,46]. These findings reaffirm HA’s critical role in improving alveolar ridge preservation and broader bone regeneration strategies. Future research should focus on standardizing HA formulations and exploring their synergistic effects with diverse grafting techniques.

However, further studies, with larger and more diverse samples and longer follow-up periods, should be conducted in the future to consolidate the currently available results and to further investigate the role of hyaluronic acid as an adjunct in bone regeneration following socket preservation.

## 5. Conclusions

The application of HA in ARP protocols demonstrates significant potential for optimizing clinical and regenerative outcomes. Evidence from this systematic review highlights the ability of HA, particularly when combined with grafting materials, to enhance bone regeneration, reduce resorption, and stabilize grafts. These findings underscore its dual role as a biological and structural adjunct in oral regenerative procedures. However, variations in study designs, sample sizes, and HA formulations necessitate further well-designed clinical trials with standardized methodologies and larger cohorts to validate and extend these results. Future research should also explore the synergistic potential of HA with advanced biomaterials to refine and optimize alveolar ridge preservation techniques.

## Figures and Tables

**Table 1:** Search strategy.

PubMed (“hyaluronic acid” [ALL FIELDS] AND “alveolar ridge preservation”) (“hyaluronic acid”[MeSH Terms] OR (“hyaluronic”[All Fields] AND “acid”[All Fields]) OR “hyaluronic acid”[All Fields]) AND (“alveolar process”[MeSH Terms] OR (“alveolar”[All Fields] AND “process”[All Fields]) OR “alveolar process”[All Fields] OR (“alveolar”[All Fields] AND “ridge”[All Fields]) OR “alveolar ridge”[All Fields]) AND (“preservation, biological”[MeSH Terms] OR (“preservation”[All Fields] AND “biological”[All Fields]) OR “biological preservation”[All Fields] OR “preservation”[All Fields] OR “preserved”[All Fields] OR “preservations”[All Fields] OR “preserve”[All Fields] OR “preserves”[All Fields] OR “preserving”[All Fields])
Scopus (hyaluronic AND acid AND alveolar AND ridge AND preservation)
Web of Science (“hyaluronic acid” [ALL FIELDS] AND “alveolar ridge preservation”)
Lilacs (“hyaluronic acid” [ALL FIELDS] AND “alveolar ridge preservation”)

**Table 2:** Summary of included studies in the systematic review: year, number, and age of patients, methods, and results.

Author	Year	Number and Age of Patients	Methods	Results
Abaza et al. [3]	2023	36 patients (20 females and 16 males), with age between 20 and 50 years old	Injectable platelet-rich fibrin versus hyaluronic acid with bovine-derived xenograft	ARP with injectable platelet-rich fibrin or hyaluronic acid mixed with xenografts reduces changes after tooth extraction.
Husseini et al. [22]	2023	7 patients, with age between 18 and 60 years old	Cross-linked hyaluronic acid addition in demineralized bovine bone	The combination of cross-linked hyaluronic acid with demineralized bovine bone resulted in significantly lower volumetric resorption (26.96%) and linear resorption rate (0.73 mm) compared to using demineralized bovine bone alone (36.56% and 1.42 mm).
Kloss et al. [23]	2024	40 patients (21 males and 19 females) between 34 and 64 years old	Hyaluronic acid-enriched allografts	Adding hyaluronic acid to allogeneic bone grafting material improved outcomes in preserving extraction sockets.
Eeckhout et al. [24]	2021	20 patients (7 males, 13 females) at least 18 years old	Hyaluronic acid gel as a wound-healing agent	This randomized controlled trial found no significant differences in wound dimensions, patient-reported outcomes, clinical outcomes, or soft tissue changes between alveolar ridge preservation sites treated with hyaluronic acid gel and those treated without it.

## Data Availability

Dataset available upon request from the authors to the corresponding author.

## Abbreviations

The following abbreviations are used in this manuscript:

ARP	Alveolar Ridge Preservation
HA	Hyaluronic Acid
PECO	Population, Exposure, Comparator, Outcomes
PRISMA	Preferred Reporting Items for Systematic Reviews and Meta-Analyses
ROBINS-E	Risk of Bias in Non-randomized Studies of Interventions
RCT	Randomized Controlled Trial
I-PRF	Injectable Platelet-Rich Fibrin
BMP-2	Bone Morphogenic Protein-2
HU	Hounsfield Units
CBCT	Cone-Beam Computed Tomography
